# Serologic Markers for Detecting Malaria in Areas of Low Endemicity, Somalia, 2008

**DOI:** 10.3201/eid1603.090732

**Published:** 2010-03

**Authors:** Teun Bousema, Randa M. Youssef, Jackie Cook, Jonathan Cox, Victor A. Alegana, Jamal Amran, Abdisalan M. Noor, Robert W. Snow, Chris Drakeley

**Affiliations:** London School of Hygiene and Tropical Medicine, London, UK (T. Bousema, J. Cook, J. Cox, C. Drakeley); University of Alexandria, Alexandria, Egypt (R.M. Youssef); Kenya Medical Research Institute–Wellcome Trust Research Programme, Nairobi, Kenya (R.M. Youssef, V.A. Alegana, J. Amran, A.M. Noor, R.W. Snow); Roll Back Malaria–World Health Organization, Hargeisa, Somalia (J. Amran); University of Oxford, Oxford, UK (A.M. Noor, R.W. Snow)

**Keywords:** Plasmodium falciparum, Plasmodium vivax, transmission, malaria, parasites, serology, immunity, heterogeneity, Somalia, research

## Abstract

Areas in which malaria is not highly endemic are suitable for malaria elimination, but assessing transmission is difficult because of lack of sensitivity of commonly used methods. We evaluated serologic markers for detecting variation in malaria exposure in Somalia. *Plasmodium falciparum* or *P*. *vivax* was not detected by microscopy in cross-sectional surveys of samples from persons during the dry (0/1,178) and wet (0/1,128) seasons. Antibody responses against *P. falciparum* or *P. vivax* were detected in 17.9% (179/1,001) and 19.3% (202/1,044) of persons tested. Reactivity against *P. falciparum* was significantly different between 3 villages (p<0.001); clusters of seroreactivity were present. Distance to the nearest seasonal river was negatively associated with *P. falciparum* (p = 0.028) and *P. vivax* seroreactivity (p = 0.016). Serologic markers are a promising tool for detecting spatial variation in malaria exposure and evaluating malaria control efforts in areas where transmission has decreased to levels below the detection limit of microscopy.

## Abstract 2

These markers can identify spatial variations in transmission patterns.

Sub-Saharan Africa has the highest incidence of malaria caused by *Plasmodium falciparum*. Almost all areas where *P*. *falciparum* parasite prevalence is >50% in the general population are located in Africa (*1*). However, malaria is not uniformly distributed (*1**,**2*) and many parts of Africa are characterized by low transmission intensity of malaria (*1*). These areas are considered suitable for intensive malaria control and disease elimination (*3**,**4*).

Assessing malaria transmission intensity and evaluating interventions are complicated at low levels of malaria transmission. Assessing transmission intensity directly by determining the exposure to malaria-infected mosquitoes (entomologic inoculation rate [EIR]) is difficult when mosquito numbers are low, sometimes below the detection limits of commonly used trapping methods (*5**,**6*), and spatial and temporal variations in mosquito densities necessitate long-term intensive sampling (*5**,**7**,**8*). Determination of malaria parasite prevalence in the human population is a commonly used alternative (*9*), but it also becomes less reliable as an indicator of transmission intensity when endemicity is low (*3*,*9*,*10*). Therefore, an alternative method is needed to assess transmission intensity, evaluate interventions, and obtain information for control programs in areas of low endemicity.

Prevalence of antibodies against malaria parasites has been explored as a means of assessing malaria transmission intensity (*11*–*13*). Antibody seroconversion rates are less susceptible to seasonal fluctuations in malaria exposure (*11*,*12*), show a tight correlation with EIR (*12*,*13*), and show potential to detect recent changes in malaria transmission intensity (*14*). Serologic markers could be particularly useful in areas of low endemicity, where it may be easier to detect relatively long-lasting antibody responses than a low prevalence of malaria infections in the human population or infected mosquitoes. We used serologic markers of exposure to determine spatial variation in malaria transmission intensity in an area of low endemicity in Somalia (*15*).

## Methods

### Study Area

This study was conducted in the Gebiley District in Somaliland in northwestern Somalia. The district has a predominantly arid landscape with a few seasonal rivers and patches of irrigated farmlands. It is an area of intense seasonal rainfall with an average annual precipitation of 59.9 mm (2004–2007) and 2 peaks in rainfall in April and August. Three moderately sized communities were randomly selected from census maps by using spatial random sampling techniques in Arcview version 3.2 (Environmental Systems Research Institute, Redlands, CA, USA) (*16*). These communities were the villages of Xuunshaley (9.72140°N, 43.42416°E), Badahabo (9.68497°N, 43.65616°E), and Ceel-Bardaale (9.81777°N, 43.47455°E). The research protocol was reviewed and approved by the Research Ethics Review Committee of the World Health Organization (RPC246-EMRO) and the Ethical Committee of the Ministry of Health and Labor, Republic of Somaliland.

### Data Collection

Two cross-sectional surveys were conducted. The first survey was conducted in March 2008 to determine parasite carriage at the end of the dry season (*16*). The purpose of the survey and the procedures were first discussed with the clan elders; thereafter, each household was visited, and informed consent was sought from each head of household. Households that agreed to participate were geolocated by using a global positioning system (Garmin eTrex; Garmin International, Inc., Olathe, KS, USA), and information was collected on demographic characteristics, bed net use, and travel history of the participants. Distance to seasonal rivers or other water bodies and distance to the nearest livestock enclosure was determined by using the global positioning system.

Individual written consent was obtained from all literate adults; illiterate adults provided consent by a thumbprint in the presence of an independent literate adult witness. For children <18 years of age, consent was obtained from parents or guardians, and children 12–18 years of age who could not write also provided consent by a thumb print.

One fingerprick blood sample was obtained from each respondent for the preparation of a *P*. *falciparum* antigen–specific rapid diagnostic test (RDT) (Paracheck-Pf; Orchid Biomedical Systems, Goa, India) sample and thick and thin blood smears. One-hundred high-power microscopic fields were examined and an additional 100 fields were examined if the first 100 fields were negative. RDT results were used for treatment with sulfadoxine-pyrimethamine and 3 doses of artesunate according to national guidelines. A second cross-sectional survey was conducted at the end of the wet season (August–September 2008) by using procedures identical to those described above, except that part of the fingerprick blood sample was placed on filter paper (3 MM; Whatman, Maidstone, UK) as described by Corran et al. (*17*).

### Entomologic Surveys

Presence of *Anopheles* spp. mosquitoes in the area was determined by larvae collections in all permanent water bodies (artificial rain water reservoirs, wells, boreholes, stagnant storage pits, and riverbeds) in the 3 villages at the end of the wet season. Locally produced 250-mL dippers with a white surface were used. Five to 10 dips were made in the large water bodies and the presence of *Anopheles* spp. larvae was visually assessed and recorded.

### Elution of Serum

Filter paper samples were stored at 4°C with desiccant until processed. A 3.5-mm blood spot, equivalent to ≈3 μL of blood (*17*), was punched from the filter paper and placed in a labeled well of a low-binding 96-well titer plate. A total of 300 μL of reconstitution buffer (phosphate-buffered saline [PBS], 0.05%Tween, and 0.1% [wt/vol] sodium azide) was added, and plates were sealed and rocked gently at room temperature overnight and subsequently stored at 4°C. The reconstituted blood spot solution was equivalent to a 1:100 dilution of whole blood or a 1:200 dilution of serum.

### ELISAs

All reconstituted filter paper spots were tested at a final serum dilution equivalent of 1:1,000 for human immunoglobulin G antibodies against *P*. *falciparum* merozoite surface protein 1_19_ (MSP-1_19_) and 1:2,000 for antibodies against apical membrane antigen 1 (AMA-1) by using described ELISA methods (*12*,*17*). Briefly, recombinant MSP-1_19_ (Wellcome genotype) and AMA-1 (3D7) were coated overnight at 4°C at a concentration of 0.5 μg/mL. Plates were washed by using PBS, 0.05% Tween 20 (PBS/T) and blocked for 3 h with 1% (wt/vol) skim milk powder in PBS/T. Positive controls (a pool of hyperimmune serum) and negative controls (European malaria-negative volunteers) were added in duplicate to each plate. The plates were washed and horseradish peroxidase–conjugated rabbit anti-human immunoglobulin G (Dako, Roskilde, Denmark) (1:5,000 dilution in PBS/T) was added to all wells. Plates were developed for 20 min by using an *o*-phenylenediamine dihydrochloride substrate solution. Reactions were stopped with 2 mol/L H_2_SO4. Plates were read immediately at 492 nm and optical density (OD) values recorded. For *P*. *vivax*, an identical protocol was used with MSP-1_19_ (0. 5 μg/mL) (*18*) and AMA-1 (0. 5 μg/mL). Serum in this protocol was used at 1:1,000 dilutions for both antigens.

### Data Management and Statistical Analyses

Data were double-entered and imported into STATA version 10 (StataCorp LP, College Station, TX, USA). Duplicate OD results were averaged and normalized against the positive control sample on each plate. A cutoff value above which samples were considered antibody positive was defined by using a mixture model as described (*17*). Distribution of normalized OD values was fitted as the sum of 2 Gaussian distributions by using maximum-likelihood methods. The mean OD of the Gaussian distribution corresponding to the seronegative population plus 3 SD values was used as the cutoff value for seropositivity (J. Cook et al., unpub. data). A separate cutoff value was generated for each antigen (MSP-1_19_ and AMA-1) for each species (*P*. *vivax* and *P*. *falciparum*). The seroconversion rate was estimated by fitting a simple reversible catalytic model to the measured seroprevalence by age in years by using maximum-likelihood methods. The serologic-derived annual EIR was then estimated by using the MSP-1_19_ seroconversion rate and a calibration curve derived from determined values (*11*).The titer of antibody responses was estimated by using the formula dilution/[maximum OD/(OD test serum – minimum OD) – 1]; the median titer and interquartile range (IQR) are given. Because of low overall antibody prevalence, antibody responses were combined by species to determine the presence of any reactivity against *P*. *falciparum* or *P*. *vivax*. As a quantitative measure of reactivity to either malaria species, the highest titer in the MSP-1_19_ and AMA-1 ELISAs was used.

Factors associated with *P*. *falciparum* or *P*. *vivax* seroreactivity were determined for each village separately by using generalized estimating equations adjusting for correlation between observations from the same household. The following factors were tested in the models: age in years, distance to the nearest seasonal river (in 100 m), distance to the nearest enclosure of livestock (in 100 m), number of household members, number of houses in a 100-m radius, roofing material, wall material, floor material, travel history, recent or regular bed net use, and an indicator of household wealth. The household wealth index was calculated on the basis of principal component analysis on characteristics such as ownership of a television, radio, telephone, bicycle, motorbike, cattle, and access to electricity (*19*). Variables that were significant at p = 0.10 in univariate analyses were added to the multivariate model and retained in the final multivariate model if their association with immune responses was statistically significant at p<0.05.

For detection of spatial clusters in immune responses, age-adjusted log_10_-transformed ODs were calculated as described by Wilson et al (*20*). First, Loess lines were fitted to scatter plots of age against log-transformed ODs for each antigen separately. For *P*. *falciparum* MSP-1_19_ and *P*. *vivax* AMA-1, the linear regression was split at 49 and 46 years of age. Log-transformed ODs were adjusted for age by linear regression. SaTScan software (*21*) was used for detection of spatial clustering in log-transformed age-adjusted OD values by using the normal probability model. A circular-shaped window was used to systematically scan the area of each village separately; statistical significance of the clusters was explored by using 999 Monte Carlo replications to ensure adequate power for defining clusters. The upper limit was specified as 50% of the village population. Significant increases in ODs were detected by calculation of the likelihood ratio for each window. Only clusters were reported that appeared for MSP-1_19_, AMA-1, and their combined age-adjusted ODs. Maps were made by using ArcGIS version 9.1 (Environmental Systems Research Institute).

## Results

The 2 cross-sectional surveys were completed in March (dry season, n = 1,178) and August–September (wet season, n = 1,128) 2008. These surveys were characterized by a clear seasonality with no rainfall detected during November 2007–March 2008 and a median monthly rainfall of 114.5 mm in April–August 2008. None of the survey participants were positive by rapid diagnostic test, and *P*. *falciparum* or *P*. *vivax* parasites were not detected on any of the examined blood slides (Table 1). Available hospital records indicated 2/283 slide-confirmed, RDT-confirmed malaria cases in the study area in July and August 2008 (T. Bousema, unpub. data). Travel history was not available for these persons. During August–September 2008, a total of 464 potential breeding sites were examined in Xuunshaley (n = 40), Badahabo (n = 42), and Ceel-Bardaale (n = 382). In Ceel-Bardaale, 158 *Anopheles* mosquito larvae were found at 81 of 382 examined sites. In the 2 other villages, no *Anopheles* larvae were observed.

**Table 1:** Characteristics of persons included in cross-sectional survey for *Plasmodium falciparum* and *P. vivax* infection, Somalia, 2008*

Characteristic	Dry season, Mar–Apr	End of wet season, Aug–Sep
No. persons	1,178	1,128
Age, y, median (IQR)	17 (6–36)	15 (6–37)
Female	48.6 (573/1,178)	50.8 (573/1,128)
Reported regular bed net use	1.9 (22/1,158)	2.2 (25/1,128)
Reported fever in 14 d preceding survey	4.8 (57/1,179)	0.6 (7/1,128)
Temperature >37.5°C at time of survey	0.8 (10/1,177)	1.1 (12/1,124)
Positive rapid diagnostic test result	0 (0/1,173)	0 (0/1,106)
*Plasmodium falciparum* parasite prevalence†	0 (0/1,173)	0 (0/1,106)
*P. vivax* parasite prevalence†	0 (0/1,173)	0 (0/1,106)

*IQR, interquartile range (25th−75th percentile). Values are % (no. positive/no. tested) unless otherwise indicated. †Determined by screening 200 high-power microscopic fields.

### Malaria Exposure Assessed by Immunologic Methods

In August–September 2008, serum samples were collected from 1,128 persons in Xuunshaley (n = 271), Badahabo (n = 160), and Ceel-Bardaale (n = 697) (Table 2). In the 3 months before the survey, 19 persons reported having traveled to areas that are known to have higher malaria endemicity for a median of 4 (IQR 2–20) days. Persons who reported traveling to areas highly endemic for malaria were more likely to have a positive response to *P*. *falciparum* (odds ratio [OR] 2.62, 95% confidence interval [CI] 0.98–7.01, p = 0.054) but not to *P*. *vivax* (OR 1.18, 95% CI 0.42–3.32, p = 0.75), after adjustment for age and village of residence. These 19 persons were excluded from further analyses.

**Table 2:** Immune responses against *Plasmodium falciparum* and *P. vivax* in study participants, by village, Somalia, 2008*

Characteristic	Village†			p value‡
	Xuunshaley	Badahabo	Ceel-Bardaale	
No. persons	271	160	697	
Median age, y (IQR)	20 (7–40)	17.5 (5–35)	13 (6–35)	0.04
*P. falciparum* immune response				
Combined	9.4 (23/244)	21.7 (30/138)	20.4 (126/619)	<0.001
MSP-1	5.1 (13/254)	13.4 (19/142)	15.0 (95/634)	<0.001

	251.2 (155.0–285.3)	214.8 (169.8–275.5)	248.5 (190.5–397.2)	0.12
AMA-1	4.8 (12/252)	9.2 (13/141)	8.9 (58/653)	0.02

	169.1 (137.0–190.9)	189.3 (158.4–225.5)	233.7 (170.7–487.0)	0.12
*P. vivax* immune response				
Combined	16.1 (40/248)	21.0 (31/148)	20.2 (131/648)	0.33
MSP-1	11.9 (30/252)	13.9 (21/151)	10.4 (58/648)	0.33

	333.9 (271.4–463.9)	342.4 (280.1–374.8)	291.5 (248.7–393.3)	0.39
AMA-1	5.2 (13/252)	7.4 (11/149)	12.8 (85/665)	0.001
	151.5 (146.0–202.1)	183.5 (155.7–275.7)	227.1 (184.6–390.8)	0.10

*IQR, interquartile range (25th–75th percentile); MSP-1, merozoite surface protein 1; AMA-1, apical membrane antigen 1. †Values are % prevalence (no. positive/no. tested) or approximate median titer (IQR) only for seropositive persons unless otherwise indicated. ‡Adjusted for age and correlations between observations from the same household, when applicable.

**Figure 1:**
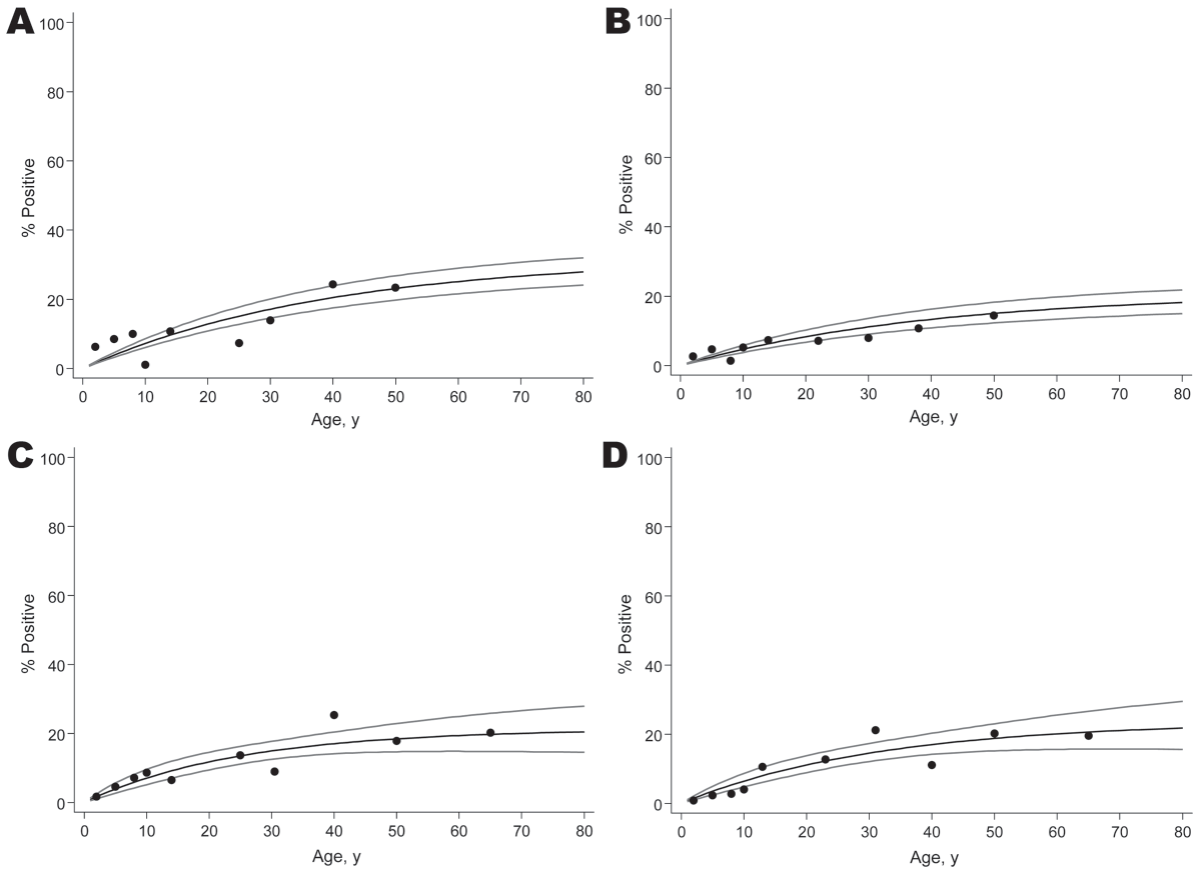
Seroprevalence data for antibodies against A) *Plasmodium falciparum* merozoite surface protein 1_19_ (MSP-1_19_), B) *P. falciparum* apical membrane antigen 1 (AMA-1), C) *P. vivax* MSP-1_19_, and D) *P. vivax* AMA-1 by age in the study population, Somalia, 2008. Gray lines indicate 95% confidence intervals. Seroconversion rates (95% confidence intervals) were as follows: *P. falciparum* MSP-1_19_ 0.0082 (0.0068–0.097); AMA-1 0.0053 (0.0042–0.0066); *P. vivax* MSP-1_19_ 0.0086 (0.0055–0.0133); AMA-1 0.0075 (0.0050–0.0112).

All antigens tested showed a clear increase in seroprevalence with a person’s age (Figure 1). The data did not suggest a recent reduction in malaria transmission intensity (*14*). The EIR for *P*. *falciparum* based on seroconversion rates for MSP-1_19_ and AMA-1 (*11*) was <0.1 infectious bites/person/year. When MSP-1_19_ and AMA-1 data were combined, 17.9% (179/1,001) of the persons tested showed reactivity against *P*. *falciparum* (i.e., had antibodies against *P*. *falciparum* MSP-1_19_, AMA-1, or both) and 19.3% (202/1,044) against *P*. *vivax*. There was a significant positive association between reactivity against *P*. *falciparum* and *P*. *vivax* (p<0.001). However, only 39.8% (66/166) of persons with antibodies against *P*. *falciparum* also responded against *P*. *vivax* antigens, and there was no apparent correlation between antibody titers against antigens of the 2 malaria species (p>0.58).

### Spatial Patterns in Seroreactivity

*P*. *falciparum* antibody prevalence was 9.4% (23/244) in Xuunshaley, 21.7% (30/138) in Badahabo (p = 0.001), and 20.4% (126/619) in Ceel-Bardaale (p<0.001) (Table 2). *P*. *vivax* antibody prevalence was 16.1% (40/248) in Xuunshaley, 21.0% (31/148) in Badahabo (p = 0.11), and 20.2% (131/648) in Ceel-Bardaale (p = 0.13) (Table 2).

**Figure 2:**
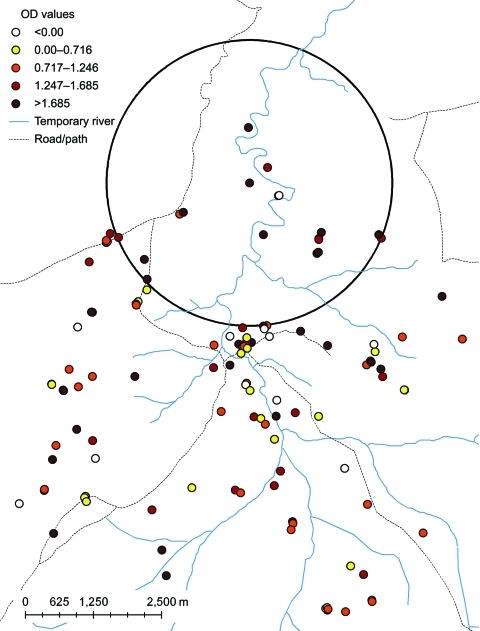
Age-adjusted optical density (OD) values for antibodies against *Plasmodium falciparum* in the study population, Ceel-Bardaale, Somalia, 2008. Colored dots indicate mean age-adjusted optical densities per household for combined seroreactivity to *P. falciparum* merozoite surface protein 1_19_ and apical membrane antigen 1. The large circle indicates a statistically significant cluster of higher *P. falciparum* seroreactivity that was detected by a spatial scan on the age-adjusted seroreactivity of individual study participants to both *P. falciparum* antigens (p = 0.002). As a result of age adjustment, some persons had lower than expected seroreactivities. This adjustment resulted in negative OD values.

Age-adjusted *P*. *falciparum* seroreactivity was significantly increased in a cluster of 18 households (108 persons) in Ceel-Bardaale (p = 0.002) (Figure 2). In Xuunshaley, there was a small cluster of 6 households (27 persons) with a higher age-adjusted *P*. *vivax* seroreactivity (p = 0.005).

### Factors Associated with Seroreactivity

Seroreactivity data were analyzed for villages separately because villages were >7 km apart and were therefore likely to have their own transmission characteristics. In all 3 villages, *P*. *falciparum* antibody prevalence increased with age (Table 3). For Ceel-Bardaale, an independent negative association was found between *P*. *falciparum* antibody responses and distance to the nearest seasonal river (OR 0.94, 95% CI 0.88–0.99, p = 0.03) after adjustment for age and correlation between observations from the same household. Within the group of persons who had a positive antibody response against *P*. *falciparum*, the titer increased with age in Xuunshaley (β = 1.74, SE = 0.81, p = 0.031) and Ceel-Bardaale (β = 11.48, SE = 3.49, p = 0.001).

**Table 3:** Factors associated with *Plasmodium falciparum* or *P. vivax* seroprevalence in 3 villages, Somalia, 2008*

Village	Factor	*P. falciparum*			*P. vivax*	
		OR (95% CI)	p value		OR (95% CI)	p value
Xuunshaley	Age	1.02 (1.00–1.04)	0.029		1.04 (1.02–1.06)	<0.001
Badahabo	Age	1.03 (1.01–1.05)	0.002		1.03 (1.01–1.05)	0.006
Ceel-Bardaale	Age	1.03 (1.02–1.04)	<0.001		1.03 (1.02–1.04)	<0.001
	Distance to river†	0.94 (0.88–0.99)	0.028		0.93 (0.88–0.99)	0.016

*OR, odds ratio; CI, confidence interval. Estimates are adjusted for correlation between observations from the same household. †Nearest seasonal river.

Similar to *P*. *falciparum*, *P*. *vivax* antibody prevalence increased with age in all 3 villages (Table 3). For Ceel-Bardaale, distance to the nearest seasonal river was negatively associated with *P*. *vivax* immune response (OR 0.93, 95% CI 0.87–0.99, p = 0.02) after adjustment for age and correlation between observations from the same household. *P*. *vivax* antibody titer did not increase with age or any other factor in those persons who were seropositive. Household factors, socioeconomic factors, distance to the nearest livestock enclosure, and use of mosquito netting were not independently associated with immune responses against *P*. *falciparum* or *P*. *vivax*.

Although seroprevalence and antibody titers were higher in older age groups, seroreactivity was also observed in young children. *P*. *falciparum* antibodies were detected in 22 children <5 years of age (median titer 216.5, IQR 173.2–248.5); 10 had antibodies against *P*. *vivax* (median titer 220.1, IQR 190.4–262.4), and 2 of these children had antibodies against *P*. *falciparum* and *P*. *vivax*. Thirty children <5 years of age who responded to malaria antigens were from all 3 villages (3 from Xuunshaley, 7 from Badahabo, and 20 from Ceel-Bardaale). Travel to areas in which malaria was highly endemic in the past 3 months was not reported for any of these children with antibodies against *P*. *vivax*, *P*. *falciparum*, or both. In children <5 years of age, a response against *P*. *falciparum* antigens was not related to a response against *P*. *vivax* antigens (p = 0.30).

## Discussion

We showed that serologic markers can be used to detect heterogeneity in malaria transmission in the Gebiley District of Somalia where malaria transmission occurs at levels too low to be detected by microscopy. None of the slides or rapid diagnostic tests showed parasite carriage in the population, and MSP-1_19_ and AMA-1 seroprevalence data showed a clear increase in seroreactivity with age and evidence for variation in exposure to malaria between and within villages.

Malaria is perceived as a public health problem in the study area (*22*), and the 2 slide-confirmed malaria cases confirm local clinical malaria episodes. Malaria transmission in the Gebiley District could not be confirmed by microscopy or RDT in 2 large cross-sectional surveys in the general population. However, our serologic findings confirmed the occurrence of malaria transmission in the area. Using a validated model to relate age-specific seroconversion rates to EIR (*11*), we estimated that *P*. *falciparum* transmission intensity in this area in Somalia was low (EIR <0.1 infectious bites/person/year). Because of the longevity of antibody responses, this estimate should be interpreted as an average EIR experienced over several years. The low EIR appeared to be supported by examination of breeding sites at the end of the wet season, which confirmed the presence of malaria vectors at a low density. We did not directly determine the EIR by sampling adult mosquitoes because the low density of mosquitoes would have required intensive sampling over different seasons (*6*,*23*).

Serologic data showed a clear age-dependency in malaria-specific immune responses, which suggested exposure-driven age acquisition of antibody response. Once acquired, antibody responses to MSP-1_19_ and AMA-1 will persist for several years (L.C. Okell, unpub. data) (*12*), and the rate of acquisition in younger age groups is therefore critical for determining current malaria transmission intensity. The maximum seroprevalence for individual malaria antigens did not exceed 25% in the oldest age groups, which is comparable to areas of low malaria endemicity in northeastern Tanzania (*12*).

Because of the longevity of antibody responses, seroreactivity may not necessarily be the result of recent exposure or exposure in the study area (*11*,*24*–*26*). Considerable changes in transmission intensity in the study area would have been detected by the model (*14*). However, especially in adults, exposure to parasites earlier in life and a history of traveling to malaria-endemic areas can obscure immune responses resulting from recent local transmission (*25*). Our data indicate that although antibodies may have been acquired outside the study area, ongoing local malaria transmission at a low intensity is likely. Elimination of false-positive results to reliably detect low-level local malaria transmission is necessary.

Cross-reactivity between immune responses to malaria and other parasites have been reported (*27*,*28*) but are expected to be more pronounced when whole parasite extract is used instead of recombinant proteins representing single antigens. The chance of cross-reactive antibody responses may be minimized by using sera at a minimum dilution of 1:80 (*27*). Our serum samples were tested at considerably higher dilutions and we observed no relation in antibody titers between the homologous antigens of *P*. *falciparum* and *P*. *vivax*. Moreover, our method for calculating seropositivity derives its seronegative population from within the study sample, thereby minimizing bias caused by local cross-reactive antigens. Although this method does not rule out cross-reactive antigens, it makes it unlikely. Antibody responses in young children who are unlikely to have acquired infections outside the study area, and for whom no recent travel history was reported, also suggest recent malaria transmission. In our study area, several children <5 years of age had antibody titers >200 to *P*. *falciparum* (n = 17) or *P*. *vivax* (n = 6). The presence of strong antibody responses (indirect fluorescent antibody titer >20) in children <15 years of age was used as evidence for active transmission of malaria in area of low endemicity in Middle America (Costa Rica) (*25*,*26*).

The indication for local malaria transmission we provide in this study is relevant for local health workers who should be prepared for fever investigations with standard parasitologic techniques (microscopy and RDT). Malaria should be considered as a plausible cause of febrile illness, particularly in an epidemic form. Low-intensity malaria transmission and the presence of malaria vectors make the area susceptible to malaria epidemics, which can have a high mortality rate in resource-poor areas (*29*), especially if outbreak detection systems (*30*) are not feasible because of a poor health infrastructure.

We observed heterogeneity in seroreactivity within the study area. Although the 3 villages had low transmission intensity and showed no difference in microscopic parasite carriage, serologic markers showed variation in malaria exposure. Antibody prevalence against *P*. *falciparum* and, less markedly, *P*. *vivax* were lowest in Xuunshaley, which was furthest from seasonal rivers. Combined *P*. *falciparum* MSP-1_19_ and AMA-1 antibody prevalence was 2× higher in Badahabo and Ceel-Bardaale than in Xuunshaley. SaTScan analysis indicated heterogeneity in malaria exposure at a microepidemiologic level. We observed 1 statistically significant cluster of persons with higher seroreactivity against *P*. *falciparum* and 1 with higher seroreactivity against *P*. *vivax*. In Ceel-Bardaale, where households were scattered along a delta of seasonal rivers, antibody prevalences to *P*. *falciparum* and *P*. *vivax* were negatively associated with distance to the nearest river. In several areas of higher endemicity, distance to the nearest body of water has been related to malaria incidence (*5*,*20*,*31*,*32*) and immune responses (*20*,*32*). No other factors were significantly related to malaria-specific immune responses.

Our data indicate that serologic markers can be used to determine variation in transmission intensity at levels of malaria transmission that are too low for sensitive assessments by microscopy, RDT, or entomologic tools. The sensitivity of serologic analysis to detect small-scale differences in transmission intensity may prove extremely useful in evaluating malaria control programs in areas where conventional malariometric markers fail. It may also provide vital information on which areas are most likely to be receptive to transmission if malaria epidemics were to occur.
